# HPTLC Method for the Simultaneous Estimation of Emtricitabine and Tenofovir in Tablet Dosage Form

**DOI:** 10.4103/0250-474X.51951

**Published:** 2009

**Authors:** Maithilee Joshi, A. P. Nikalje, M. Shahed, M. Dehghan

**Affiliations:** Y. B. Chavan College of Pharmacy, Dr. Rafiq Zakaria Campus, Rauza Bagh, Auranagabad-431 001, India

**Keywords:** HPTLC analysis, emtricitabine, tenofovir

## Abstract

A simple, precise, accurate and rapid high performance thin layer chromatographic method has been developed and validated for the estimation of emtricitabine and tenofovir simultaneously in combined dosage form. The stationary phase used was precoated silica gel 60F 254. The mobile phase used was a mixture of chloroform: methanol (9:1 v/v). The detection of spots was carried out at 265 nm. The method was validated in terms of linearity, accuracy, precision and specificity. The calibration curve was found to be linear between 200 to 1000 ng with regression coefficient of 0.9995. The proposed method can be successfully used to determine the drug content of marketed tablet formulation.

The combination of emtricitabine (EMT) and tenofovir (TEN) has recently been introduced in the market. Chemically emtricitabine[1] is 4-amino-5-fluoro-1-[2-(hydroxymethyl)-1,3-oxathiolan-5-yl]- pyrimidin-2-one. Emtricitabine is a nucleoside reverse transcriptase inhibitor (NRTI) for the treatment of HIV infection in adults. Tenofovir[2] is 1-(6-aminopurin-9-yl) propan-2-yl-oxymethylphosphonic acid. It is nucleotide analogue reverse transcriptase inhibitor (NRTI). Literature survey reveals that, so far no HPTLC method has been reported for the simultaneous estimation of EMT and TEN in formulation. There is only one HPLC method reported[3] for this combination and it requires solid phase extraction of drugs from plasma; the total run time for this method is 18 min, which is quite a long time. Several methods were reported[4,5] for the individual estimation of EMT and TEN but not for the combined form. If the reported individual methods are applied for the analysis of the tablets containing EMT and TEN, it would require double time for analysis; the method would not be rapid, less expensive, whereas the developed HPTLC method for simultaneous estimation of the ingredients of tablets would save analysis time and economy. In the present investigation, an attempt has been made to develop a rapid, accurate, precise and cost effective HPTLC method for simultaneous estimation of EMT and TEN in combined dosage form.

EMT and TEN standards were procured as a gift samples from Cipla Pharmaceuticals Ltd. Silica gel 60 F_254_ TLC plates (20×10 cm, layer thickness 0.2 mm, E. Merck) were used as stationary phase. All chemicals and reagents used were of analytical grade and obtained from Qualigens. Marketed formulation Tenvir EM, manufactured by Cipla, India Ltd., containing EMT (200 mg) and TEN (300 mg), was used for the analysis. The instrument used in the present study was Camag HPTLC system comprising of Camag Linnomate V automatic sample applicator, Hamilton syringe (100 μl), Camag TLC Scanner 3, Camag Win CATS software, Camag Twin-trough chamber (20×10 cm). Ultrasonicator was used for extraction of the drugs from the tablets.

EMT and TEN (10 mg each) were weighed accurately, dissolved and diluted with methanol to obtain the final concentration of 0.1 μg/μl of each drug. From this solution 1ml o is diluted to 10 ml to get 100 ng/ml. Twenty tablets were weighed accurately and ground to fine powder. Weights equivalent to 10 mg of EMT and TEN were transferred to conical flask and mixed with methanol. The solution was sonicated for 15 min. The solution was centrifuged for 20 minutes and transferred to a 10 ml volumetric flask and volume was made up to 10 ml with methanol. Required dilutions were made to get 100 μg/ml of EMT and TEN.

To carry out HPTLC analysis[6], the TLC plates were prewashed with methanol. Activation of plates was done in an oven at 50° for 5 min. The chromatographic conditions maintained were precoated silica gel 60F_254_ aluminum sheets (20×10 cm) as stationary phase, chloroform:methanol (9:1 v/v) as mobile phase, chamber and plate saturation time of 30 min, migration distance allowed was 80 mm, wavelength scanning was done at 265 nm keeping the slit dimension at 6×0.45 mm. Five microlitres of standard solutions of EMT and TEN were spotted and developed at constant temperature. Wavelength was selected by scanning standard solutions of both drugs over 200 nm to 400 nm wavelengths. EMT showed maximum absorbance at 286 nm and TEN at 264 nm. Both components showed reasonably good response at 265 nm, therefore photometric measurements were performed at 265 nm in absorption mode with Camag TLC scanner 3 using Win CATS software. Aliquots of 2.0, 4.0, 6.0, 8.0, 10.0 μl of standard solutions of EMT and TEN were applied on the TLC plate (0.1 μg/ml of drug). TLC plate was dried, developed and analyzed photometrically as described earlier.

The developed method was validated[7,8] in terms of linearity, accuracy, limit of detection, intra-day and inter-day precision and repeatability of peak area measurement and the observations are reported in Table 1. Linearity was measured in concentration range of 200-1000 ng/ml. Limit of detection was found 100 ng/spot and 50 ng/spot for EMT and TEN, respectively. Limit of quantification was found 160 ng /spot for EMT and 190 ng /spot for TEN. Precision was checked by repeatability of peak area measurement and RSD values were 0.21 for EMT and 0.19 for TEN. The method is specific for the above drugs, as the excipients do not interfere with the peaks of EMT and TEN.

**TABLE 1:** METHOD VALIDATION PARAMETERS

Parameters	Values	
	
	Emtricitabine	Tenofovir
Linearity range (ng/spot)	200-1000	200-1000
Correlation coefficient (r)	0.9995	0.9996
Regression equation (y=mx+c)		
Slope	6.82	6.29
Intercept	117.3	18.81
Limit of detection	100 ng/spot	50 ng/spot
Limit of quantification	160 ng/spot	190 ng/spot
Precision (%RSD)		
Repeatability of peak area		
measurement (n=5)	0.21	0.19

Twenty tablets were weighed accurately and ground to fine powder. Weights equivalent to 10 mg of EMT and TEN, were transferred to conical flasks and mixed with methanol. The solution was sonicated for 15 min. The solution was centrifuged for 20 minutes and transferred to a 10 ml volumetric flask and volume was made up to 10 ml with methanol. Required dilutions were made to get 100 μg/ml of EMT and TEN.

Accuracy parameter was checked by recovery studies. For EMT 99.54% recovery and for TEN 99.69% recovery was observed. Four micro litres of sample solutions of the marketed formulation were spotted on to the same TLC plate and developed. The analysis was repeated in triplicate. The content of the drug was calculated from the peak areas recorded.

The mobile phase consisting of chloroform: methanol (9:1 v/v) gave R_f_ values of 0.18±0.01 and 0.48±0.01 for EMT and TEN, respectively, with dense and compact spots desired for quantification of EMT and TEN in pharmaceutical formulations. (fig. 1) Calibration curve was obtained in the range of 200-1000 ng/ml with r^2^ value of 0.9995 and 0.9996 for EMT and TEN, respectively. Drug found by the assay of tablets was 100.16% and 99.94% for EMT and TEN, respectively. Accuracy parameter was checked by recovery studies. For EMT 99.54% recovery and for TEN 99.69% recovery was observed. Limit of detection was found to be 100 ng/spot and 50 ng/spot for EMT and TEN, respectively. Limit of quantification was found to be 160 ng/spot for EMT and 190 ng/spot for TEN. Thus, this method can routinely be applied for analysis of tablets.

**Fig. 1:**
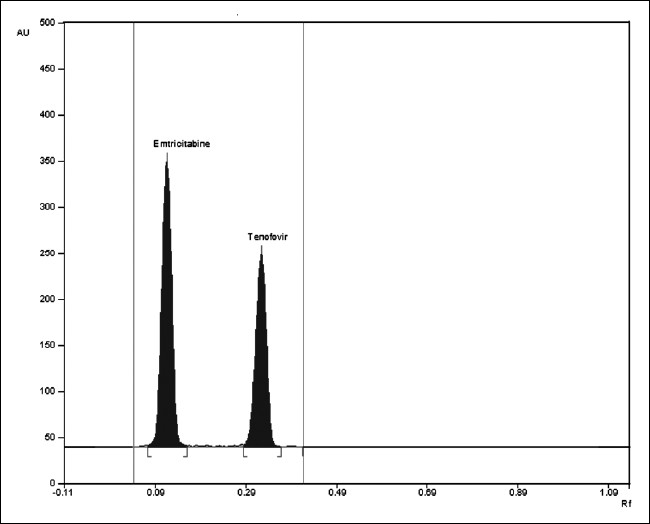
A typical HPTLC chromatogram of EMT and TEN

The linear regression data[9] (n=5) showed a good linear relationship over a concentration range of 200-1000 ng/spot and for EMT and TEN, respectively. The limit of detection for EMT was found to be 100 ng/spot and for TEN, 50 ng/spot, The intra-day precision was determined by analyzing standard solutions in the concentration range of 100 ng/spot to 500 ng/spot for EMT and TEN for 3 times on the same day, while inter-day precision was determined by analyzing corresponding standards daily for 3 day over a period of one week. Repeatability of sample application was assessed by spotting 4 μl of drug solution, 5 times on a TLC plate, followed by development of plate and recording the peak area for 5 spots. The % RSD for peak area values of EMT and TEN were found to be 0.21 and 0.19, respectively. To confirm the specificity of the proposed method, the solution of the formulation was spotted on the TLC plate, developed and scanned. It was observed that the excipients present in the formulation did not interfere with the peaks of EMT and TEN.

Recovery studies of the drugs were carried out for the accuracy parameter. These studies were carried out at three levels, i.e. multiple level recovery studies (n=3). Sample stock solution from tablet formulation of 100 μg/ml was prepared. Dilutions were made and recovery studies were performed. Percent recovery was found to be within the limits as listed in Table 2. The assay value for the marketed formulation was found to be within the limits as listed in Table 3. The low RSD value indicated the suitability of the method for routine analysis of EMT and TEN in pharmaceutical dosage forms. The developed HPTLC technique is simple, precise, specific and accurate and the statistical analysis proved that method is reproducible and selective for the analysis of EMT and TEN in bulk drug and tablet formulations.

**TABLE 2:** RECOVERY STUDIES OF EMT AND TEN

Label Claim	Total Amt. added	Amt. Recovered	% Recovery
EMT 20	1.6	1.586	99.17
	2.0	1.991	99.88
	2.4	2.385	99.59
TEN 30	2.6	2.592	99.93
	3.0	3.002	100.08
	3.4	3.368	99.07

**TABLE 3:** ANALYSIS OF EMT AND TEN

Drugs	Rf	Amount found	% drug recovered	r^2^	Standard deviation
EMT	0.18	200.33 mg	100.16	0.9995	1.70
TEN	0.47	299.84 mg	99.94	0.9996	1.77
